# Change Is Slow: Acquisition of Disability-Accessible Medical Diagnostic Equipment in Primary Care Offices over Time

**DOI:** 10.1089/heq.2023.0155

**Published:** 2024-03-07

**Authors:** Nancy R. Mudrick, Julia Blackwell, Mary Lou Breslin, Xiafei Wang

**Affiliations:** ^1^ School of Social Work, Syracuse University, Syracuse, New York, USA.; ^2^ Disability Rights Education & Defense Fund, Berkeley, California, USA.

**Keywords:** disability, medical diagnostic equipment, height-adjustable examination table, accessible weight scale, primary care

## Abstract

**Introduction:**

The lack of disability-accessible medical diagnostic equipment (MDE) in primary care impedes the receipt of quality medical care by people with mobility impairments. Cross-sectional surveys and observational studies have found <40% of medical offices have disability-accessible examination tables or weight scales. Although government agencies and advocates recommend primary care acquisition of the accessible MDE, the rate of acquisition is unknown.

**Methods:**

Using panel data, the research examined if primary care offices audited for disability accessibility increased accessible examination table and scale presence between the first and second audit. Data for 2006–2009 (Time 1 [T1]) and 2013–2016 (Time 2 [T2]) came from 1293 primary care practices associated with Medicaid managed care organizations. Permutations of presence or absence of a height-adjustable examination table and accessible weight scale were analyzed to assess rate of change across time periods.

**Results:**

More practices had disability-accessible examination tables or weight scales at the second observation, although total presence was low (12.9%, 7.9%). Practices added equipment between time periods; however, ∼60% of practices with accessible MDE at T1 no longer had it available at T2.

**Discussion:**

The acquisition rate of accessible MDE was low, despite prior auditing. Studying change in accessible MDE presence in primary care offices requires attention to equipment acquisition and its retention.

**Health Equity Implications:**

Stronger federal enforcement of Medicaid and Americans with Disabilities Act (ADA) access requirements, with regular standardized auditing of medical office accessibility, may be required to produce a more equitable health care experience for disabled people.

## Introduction

Inaccessible medical diagnostic equipment (MDE) in doctors' offices impedes receipt of quality medical care by people with mobility disability and contributes to health care delivery inequities. The scarcity of MDE that is accessible and accommodates people with mobility impairment is well documented through surveys and direct observation.^1–6^ Discussion of these barriers in publications by researchers and government agencies has occurred, but whether this has influenced primary care practices is unclear.^7–9^ The absence of accessible MDE poses significant risk to the health of patients with mobility limitation because it may cause standard elements of care to be skipped or provided in a less thorough manner.^10^

Both patients and physicians have reported that body weight was not routinely measured for patients with mobility limitations if an accessible weight scale was not available.^2,11,12^ They also reported that patients were examined seated in their wheelchairs rather than on an examination table where a height-adjustable table and/or a patient lift was not present.^1,12,13^ As well, some patients have reported they delayed initiating medical care for an emerging condition due to challenges associated with receipt of care, including concern about injury from the use of inaccessible medical examination equipment.^14^

Disability-accessible medical diagnostic equipment includes examination tables, examination chairs, weight scales, and radiology and scanning machines that safely accommodate persons with mobility limitation. The prior research on the presence of accessible MDE is based upon cross-section interviews with patients and physicians and cross-section office audits using checklists for the presence of height-adjustable examination tables, accessible weight scales, and patient lifts. Across studies conducted since 2008 that surveyed primary care practices by mail, telephone, or on-site observation, the presence of height-adjustable examination tables ranged from 10% to 40%, while the presence of accessible weight scales was 3–15%.^4–6,12,15^ What these aggregate estimates do not offer is an understanding of how quickly individual medical practices are responding to the increased attention to the need for disability-accessible MDE in doctors' offices.

The panel research reported in this study with descriptive analysis used disability accessibility data from the observation of the same primary care practice at two points in time. The overarching research aim was to assess whether primary care offices audited for disability accessibility significantly increased the presence of accessible examination tables and scales between the first and second audit. Were examination tables acquired at a different rate than accessible scales? Did sites with accessible equipment at the first audit still have that equipment at the second audit?

## Methods

### Data source

The data for this study were two waves of primary care office disability accessibility audits conducted by Medicaid managed care organizations (MMCOs) in California. The data contain only physical facility characteristics with no human subject information; therefore the Syracuse University IRB determined review was not required. The first audit, Time 1 (T1), was conducted over 2006–2009. The second audit, Time 2 (T2), occurred over 2013–2016. The accessibility audit in T1 was a voluntary add-on to a broader on-site audit that MMCOs were mandated to conduct by the State of California. In T2, the disability accessibility audit became a mandatory part of the MMCO audit of practices serving seniors and patients with disability.^16^ The audits were conducted through on-site observation by trained staff of an MMCO. MMCOs conduct the audit when a primary care practice joins the MMCO network and every 3 years thereafter. Since 2012, MMCOs have used the state-approved instrument, which is a revision of the T1 instrument.

Each MMCO holds the data it has collected, although some information is shared among the MMCOs in the same region to reduce multiple reviews of practices affiliated with more than one network. While the California Department of Health Care Services mandates the on-site audit, it does not collect the information from the MMCOs into a central database, nor require public posting of the rating of individual practice accessibility or the network collectively. Some of the MMCOs have voluntarily included information about accessibility in a practitioner's website profile or a provider directory to assist patients in the selection of a provider.

The accessibility audit instrument was developed in 2006 by two disability policy consultants and representatives from several California MMCOs. The 2006 version (T1) had 55 items; 53 elements were selected or derived from the 2004 Americans with Disabilities Act (ADA) Accessibility Guidelines (ADAAG) developed by the U.S. Access Board (https://www.access-board.gov). Two equipment questions (height-adjustable examination table and accessible weight scale) were also included, although the ADAAG did not contain any equipment standard.

The 55-item instrument was used for data collection from 2006 to 2011. In 2011, the audit tool was revised for greater clarity, expanded to 86 questions (3 with subquestions for a total of 92 measures), and updated to the 2010 ADAAG standards.^17^ The 86-question instrument (T2) included 5 equipment questions (examination table characteristics [3 questions], weight scale, and patient lift). Individual items are scored yes (present), no (absent), or does not apply. The 2011 audit instrument is available from the California Department of Health Care Services and is posted on most California MMCO websites (the 2006 instrument is available from the authors).^16^

The T1 instrument asked whether a height-adjustable table was present, which could go as low as 20 inches, but ideally as low as 17 inches. The T2 question specifies that the table should lower between 17 and 19 inches. This 2011 wording corresponds to the proposed table height standard subsequently issued by the U.S. Access Board in 2017.^18^ In addition to the 1 inch difference in the table distances from the floor, the T2 instrument asked about the space next to the height-adjustable examination table and the presence of table elements to assist transfer.

For the comparison analysis, only the single question about the presence of the height-adjustable table was used. The scale question in the T1 instrument had the words “accessible weight scale,” checked yes or no, with an explanation. The T2 question asked if a scale was available with a platform to accommodate a wheelchair or scooter, also with an explanation. Although the latter question wording is more elaborate, the explanation is identical in the T1 and T2 instruments. We judged the differences in wording sufficiently minor as to not prevent their use for comparison. Table 1 shows the wording of all the MDE questions.

**Table 1.** Comparison of T1 and T2 Question Wording for Examination Tables, Weight Scales, and Patient Lifts

2006–2009 Instrument		2013–2016 Instrument	
Evaluation criteria	Reviewer guidelines	Evaluation criteria	Reviewer guidelines
Examination tables are height adjustable, with minimum height of 20 inches (preferably 17 inches from the floor)	Self-explanatory	Is there a height-adjustable examination tables that lowers to between 17 and 19 inches from the floor to the top of the cushion?	Self-explanatory
		Is there space next to the height-adjustable examination table for a wheelchair or scooter user to approach, park, and transfer or be assisted to transfer onto the table?	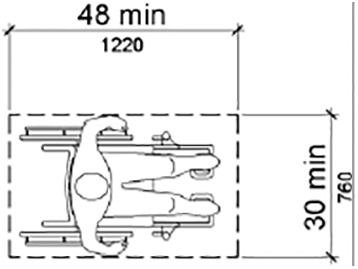
		Does the examination table provide elements to assist during a transfer (such as rails) and support a person while on the table? (if yes, please list in comments)	Items that could help support a patient while on the table would be armrests, side rails, padded straps, cushions, wedges, etc.
Accessible weight scale	Accessible scales are usable by all people, including wheelchair users, people with activity limitations, and larger people who may exceed a standard weight scale limit. This includes people with conditions that interfere with mobility, walking, climbing, using steps (joint pain, short stature, pregnancy, fatigue, respiratory and cardiac conditions, post-surgical conditions, orthopedic injuries); and/or who use mobility devices (e.g., canes, crutches, walkers)	Is a weight scale available within the medical office with a platform to accommodate a wheelchair or scooter and the patient?	Accessible scales are usable by all people, including wheelchair users, people with activity limitations, and larger people who may exceed a standard weight scale limit. This includes people with conditions that interfere with mobility, walking, climbing, using steps (joint pain, short stature, pregnancy, fatigue, respiratory and cardiac conditions, post-surgical conditions, orthopedic injuries); and/or who use mobility devices (e.g., canes, crutches, walkers).
Patient lift		Is a lift available to assist staff with transfers (portable, overhead, or ceiling mounted)?	Self-explanatory

Answers: Yes, No, N/A, not applicable. The 2006–2009 instrument is available from the authors; the 2011 instrument is at https://www.dhcs.ca.gov/formsandpubs/Documents/MMCDAPLsandPolicyLetters/PL2012/PL%2012-006.pdf

T1, Time 1; T2, Time 2.

### Study sample

In 2009, we asked the MMCOs voluntarily conducting the accessibility audit if we could analyze their data. We received audit data for 2389 practices spanning 2006–2009 from 5 of the 14 California-approved MMCOs. These were solo and group practices located in southern and central California and the San Francisco Bay Area. The number of California primary care practices in this time period is not known, preventing calculation of percentage representation.^19^ In 2017 and 2018, we requested audit data for 2013–2016 from the MMCOs. We received data from four of the MMCOs that sent 2006–2009 data. Although the datasets did not include physician or practice group name, address, practice size, or other descriptive characteristics, they did contain a Department of Health Care Services unique ID number (DHSID) and the practice's ZIP Code.

Using ID number, we were able to identify 1293 practices that appeared in the 2006–2009 (T1) and 2013–2016 (T2) datasets, enabling analysis of change in accessibility between the first and second audit. A comparison of ZIP Codes at T1 and T2 for each DHSID found different ZIP Codes for ∼2% of the providers, suggesting that nearly all practices were located in the same physical facility at the two points in time. The 1293 observations are 55.9% of the 4 MMCOs' 2313 practices present during 2006–2009. Physician retirement, practice merger, or unwillingness to continue accepting Medicaid could be reasons 1020 practices were no longer affiliated with the MMCOs.

### Measures

The key measures were the presence/absence of a height-adjustable examination table, accessible scale, and patient lift at the two time periods. All variables were in dichotomous yes/no format. The cell percentages in a 2×2 table were used to examine both movement and stasis across the two time periods.

### Data analysis

The frequency distribution of each of the three pieces of MDE at the two time periods was used to examine overall change. The cell percentages of the presence of a height-adjustable examination table and accessible weight scale were examined to see the permutations of change between T1 and T2. The statistical significance of the change was tested using McNemar's Chi-squared test, which accounts for paired observations.

## Results

The frequency distribution for the MDE questions in both time periods is displayed in Table 2. This table shows that the overall percentage of sites with a height-adjustable examination table increased by 4.8 percentage points from 8.1% to 12.9%, with a T2 total of 166 sites with an adjustable table. Within this group, 92% had the table placed with space for a patient to transfer and 25.3% had a table with additional elements to assist the patient's transfer. Table 2 shows that the accessible weight scale presence went from 3.0% to 7.9%, a similar percentage point increase as that for tables. Patient lifts were present in 3.1% of primary care office sites evaluated in both time periods. Although the total percentage of sites with a height-adjustable examination table and an accessible scale at T2 remains small, the aggregate change from T1 is statistically significant (*p*<0.001).

**Table 2.** Percentage Presence of Accessible Medical Diagnostic Equipment, T1 and T2

Accessible medical diagnostic equipment	T1: 2006–2009	T2: 2013–2016
	% Yes	% Yes
Height-adjustable examination table present^a^	8.1% (105)	12.9% (166)
Space for wheelchair or scooter next to the height-adjustable examination table	Not asked	92.4%^b^ (145)
Height-adjustable examination table has elements to assist during transfer	Not asked	25.3%^b^ (37)
Accessible weight scale present^a^	3.0% (39)	7.9% (102)
Patient lift is present	Not asked	3.1% (40)

Total *n*=1293.

^a^ *p*<0.001 Difference between T1 and T2, McNemar test for paired observations.

^b^ Of sites with a height-adjustable examination table.

The permutations of change for each site from T1 to T2 in the presence of a height-adjustable examination table and an accessible weight scale are shown in Tables 3 and 4. Table 3 shows that 82.0% had no accessible examination table across both time periods. Between T1 and T2, 9.9% of sites added a table and 3.0% with a table in T1 still had one in T2. In addition to acquisition of a height-adjustable examination table, Table 3 shows that 5.1% of sites had a height-adjustable table in T1 and no longer had one in T2. The crosstabulation of height-adjustable examination tables at T1 and T2 was statistically significant at *p*<0.001.

**Table 3.** Presence of Height-Adjustable Examination Table, 2006–2009 and 2013–2016

	Height-adjustable examination table?	
	2006–2009	2006–2009
	Yes	No
2013–2016		
Yes	3.0% (*n*=39)	9.9% (*n*=127)
2013–2016		
No	5.1% (*n*=66)	82.0% (*n*=1056)

Total number of practices with height-adjustable examination tables, using 2006 instrument=105; with 2011 instrument=166. Crosstabulation statistical significance, two-sided McNemar *p*<0.001.

**Table 4.** Presence of an Accessible Weight Scale, 2006–2009 and 2013–2016

	Accessible weight scale?	
	2006–2009	2006–2009
	Yes	No
2013–2016		
Yes	1.0% (*n*=13)	6.9% (*n*=89)
2013–2016		
No	2.0% (*n*=26)	90.0% (*n*=1158)

Total number of practices with an accessible weight scale, using 2006 instrument=39; with 2011 instrument=102. Crosstabulation statistical significance, two-sided McNemar *p*<0.001.

Table 4 shows that across the studied sites, 6.9% added an accessible scale, while 1% had a scale in both T1 and T2 time periods. Ninety percent of sites did not acquire an accessible scale between T1 and T2. A small loss of sites with accessible scales is observed with 2.0% of all sites having an accessible scale at T1, but not having one at T2. The comparison of the presence of an accessible weight scale was statistically significant (*p*<0.001, two-sided McNemar test).

We examined by MMCO what percent of all practices affiliated with that MMCO added a height-adjustable table. A range of 9.2–12.0% of an MMCO's affiliated practices added a height-adjustable examination table and 5.0–9.7% added an accessible scale. However, because some practices in the dataset are affiliated with two of the MMCOs, we could not say which MMCO may have assisted in the equipment acquisition.

## Discussion

Examination of the change in prevalence of accessible MDE in the MMCO affiliated primary care offices shows only a very modest increase in accessible equipment over a 7- to 10-year period. Overall, the presence of accessible MDE remained low. The pattern of change in the presence of a height-adjustable examination table and accessible weight scale (Tables 3 and 4) is discouraging. The number of practices with no accessible equipment in both time periods far exceeded those with changes (82% in table presence and 90% for scale). Between T1 and T2 of 1293 practices, 127 practices added a height-adjustable table and 89 added an accessible scale. This increased the total number of practices with this equipment by 61 tables and 63 scales. Which MMCO a practice was affiliated with did not appear related to whether accessible equipment was acquired, despite some MMCOs reporting they have offered financial support for accessible MDE.^20^

The total number of practices with accessible equipment would have been higher if all practices with accessible equipment at T1 still had the equipment at T2. However, 62.9% of practices with an adjustable table at T1 no longer had a qualifying table at T2. Similarly, 66.7% of practices with an accessible scale at T1 no longer had it at T2. The decrease in the presence of equipment is puzzling since one might expect that over the relatively short time period between measurements, the equipment would not deteriorate or age beyond usability.

We speculate that, for the height-adjustable examination tables, some sites had a table that met the T1 criterion, but not the 1 inch lower criterion at T2. It also is possible that the table observed at T1 was no longer in use, whether because it needed repair or had been removed. In interviews with providers about their experiences with height-adjustable examination tables, some mentioned challenges using the table in their small examination rooms.^21^ The decrease in the presence of a scale from T1 to T2 might also result from scales that malfunctioned and were not repaired, or scales moved out of the practice. Because the guidance for rating the scale question was the same across both instruments, wording differences seem an unlikely explanation for the measured decrease in scale presence.

Finally, surveyor error in observation or completion of the audit could explain some differences between time periods. While we cannot explain with certainty why a noticeable proportion of practices apparently went from having disability-accessible examination equipment to not having it, the findings point to the importance of tracking the presence of accessible equipment over time and training surveyors to identify accessible MDE correctly. Moreover, encouraging the purchase of accessible equipment is not sufficient; actions to enable their continued presence and use are necessary.

### Limitations

To our knowledge, the panel dataset used for this analysis is unique. It is derived from two on-site observations of the same primary care practice that were conducted as part of a State of California mandated audit. However, it has limitations arising from the audit instrument construction, the data collection process, and its age. The T1 and T2 instruments were devised to meet agency administrative interests, with their length and content negotiated with the MMCOs around feasibility and burden. The development was not part of a formal research project, and thus reliability assessments of data collection instruments were not conducted before fielding. The wording differences between the T1 and T2 instruments for the same element pose challenges for a totally clean comparison.

A second limitation is that the data collection process was not monitored for consistency in rater training and inter-rater reliability across the MMCO raters. The MMCOs declined to include descriptors about the practices in the data sent to us; thus, exploration of potential predictors of MDE acquisition and maintenance was not possible. Useful descriptors would have been the number of physicians in the practice, number of patients enrolled, overall patient demographics, and age and type of building in which the practice is located. Such information would have allowed exploration of whether accessible MDE is more likely to be acquired in large group practices or as practices experience a noticeable increase in their percentage of patients with mobility limitations.

Because the last observations were collected in 2016, it is likely that some medical practices added MDE since then. However, the focus here was on the rate of change. We know of no major change in policy or ADA enforcement since 2016, which might have stimulated a greater rate of MDE acquisition by MMCO primary care practices than the rate observed in this study. Finally, this dataset does not allow generalization to all primary care medical practices in California or the United States. Because the primary care office could not decline to be observed if it wished to remain part of the MMCO panel, selection bias based upon willingness to participate in the site audit is unlikely.

However, the primary care practices present in the dataset are only those affiliated with four specific Medicaid managed care plans in California that served seniors and people with disabilities. Also affecting generalization is the inability to estimate what proportion of all California primary practice sites or all Medicaid accepting practice sites are represented in our study dataset at T1. Moreover, the roles of the county, the Department Health Care Services, and MMCOs with respect to practice site monitoring may be unique to California. Medicaid accepting physician practices in other states may have different incentives to acquire accessible equipment or ways to measure equipment presence. In California and in other states, non-Medicaid accepting physicians still have disability accommodation obligations, but the oversight may be less structured.

## Health Equity Implications

It appears that the experience of a prior audit had little influence on the acquisition of accessible equipment. Growth and accountability in Medicaid managed care could motivate action because failure to provide such accommodations contributes to inequitable care.^9,22,23^ These findings should spur the U.S. Departments of Justice and Health and Human Services to engage in vigorous enforcement of the ADA and the upcoming HHS revised Section 504 MDE regulations and U.S. Access Board MDE standards.^18,24^ In addition, CMS should require on-site accessibility auditing of the primary care settings subject to disability rights laws and the Affordable Care Act.

Audits should examine sites' physical and programmatic accessibility and be conducted using a standardized instrument that has been assessed for reliability and validity. Survey raters should have consistent training, guidance, and oversight. This consistency will enable effective monitoring of changes in disability-related accessibility over time. Finally, these findings call for more panel research and for qualitative studies of equipment acquisition and use by primary care offices. These studies could offer insights useful to understanding why change has been slow, what affects decisions to acquire or not acquire accessible MDE, and the factors that influence whether the equipment remains regularly available and used so that disabled patients receive equitable health care.

## Abbreviations Used

ADA: Americans with Disabilities Act
ADAAG: Americans with Disabilities Act Accessibility Guidelines
DHSID: Department of Health Care Services unique ID number
MDE: medical diagnostic equipment
MMCO: Medicaid managed care organization
T1: Time 1
T2: Time 2
